# Single-cell RNA Sequencing Reveals Heterogeneity of Cultured Bovine Satellite Cells

**DOI:** 10.3389/fgene.2021.742077

**Published:** 2021-10-28

**Authors:** Pengcheng Lyu, Yumin Qi, Zhijian J. Tu, Honglin Jiang

**Affiliations:** ^1^ Department of Animal and Poultry Sciences, Virginia Tech, Blacksburg, VA, United States; ^2^ Department of Biochemistry, Virginia Tech, Blacksburg, VA, United States

**Keywords:** FAP, fibro-adipogenic progenitors, ScRNA-seq, cattle, skeletal muscle, myoblast

## Abstract

Skeletal muscle from meat-producing livestock such as cattle is a major source of food for humans. To improve skeletal muscle growth efficiency or quality in cattle, it is necessary to understand the genetic and physiological mechanisms that govern skeletal muscle composition, development, and growth. Satellite cells are the myogenic progenitor cells in postnatal skeletal muscle. In this study we analyzed the composition of bovine satellite cells with single-cell RNA sequencing (scRNA-seq). We isolated satellite cells from a 2-week-old male calf, cultured them in growth medium for a week, and performed scRNA-seq using the 10x Genomics platform. Deep sequencing of two scRNA-seq libraries constructed from cultured bovine satellite cells yielded 860 million reads. Cell calling analyses revealed that these reads were sequenced from 19,096 individual cells. Clustering analyses indicated that these reads represented 15 cell clusters that differed in gene expression profile. Based on the enriched expression of markers of satellite cells (PAX7 and PAX3), markers of myoblasts (MYOD1, MYF5), and markers of differentiated myoblasts or myocytes (MYOG), three clusters were determined to be satellite cells, two clusters myoblasts, and two clusters myocytes. Gene ontology and trajectory inference analyses indicated that cells in these myogenic clusters differed in proliferation rate and differentiation stage. Two of the remaining clusters were enriched with PDGFRA, a marker of fibro-adipogenic (FAP) cells, the progenitor cells for intramuscular fat, and are therefore considered to be FAP cells. Gene ontology analyses indicated active lipogenesis in one of these two clusters. The identity of the remaining six clusters could not be defined. Overall, the results of this study support the hypothesis that bovine satellite cells are composed of subpopulations that differ in transcriptional and myogenic state. The results of this study also support the hypothesis that intramuscular fat in cattle originates from fibro-adipogenic cells.

## Introduction

Single-cell RNA sequencing (scRNA-seq) is a relatively new technology that analyzes transcriptomes in individual cells by deep sequencing (Tang et al., 2009). scRNA-seq has proved to be a powerful method for assessing the heterogeneity of a cell population and for identifying rare or previously uncharacterized cell types in complex organs and tissues (Tang et al., 2009; Andrews and Hemberg, 2018).

Satellite cells are myogenic progenitor cells in skeletal muscle of postnatal animals (Mauro, 1961). Satellite cells are characterized by the expression of the transcription factor paired box protein 7 (PAX7) or PAX 3 (Maroto et al., 1997; Seale et al., 2000; Kuang and Rudnicki, 2008). Satellite cells play an essential role in skeletal muscle regeneration and growth (Dhawan and Rando, 2005). Satellite cells are normally quiescent but are activated under conditions such as muscle injury (Dhawan and Rando, 2005). Once activated, satellite cells proliferate as myoblasts, differentiate, and fuse with each other to generate muscle fibers or with existing muscle fibers to increase muscle size.

In our research involving bovine satellite cells (Ge et al., 2013; Leng et al., 2019; Leng and Jiang, 2019), we noticed that these cells in culture did not all become myofibers upon induction of myogenic differentiation. We hypothesized that bovine satellite cells are composed of subpopulations that differ in myogenic potential. The objective of this study was therefore to determine if bovine satellite cells are heterogeneous in terms of myogenic potential. We approached this objective by analyzing a population of bovine satellite cells with scRNA-seq.

## Materials and Methods

### Isolation and Culture of Bovine Satellite Cells

Bovine satellite cells were isolated from the longissimus muscle of a 2-week-old Holstein bull calf following euthanasia. Satellite cells were isolated using a procedure involving pronase digestion and differential centrifugation as described before (Hathaway et al., 1991). Satellite cells were cultured in growth medium consisting of Dulbecco’s modified eagle medium, 10% fetal bovine serum, 2 mM L-glutamine, and 1% antibiotics-antimycotics (Thermo Fisher Scientific, Waltham, MA, United States) for a week to remove dead cells and increase the number of viable cells prior to scRNA-seq. The animal-related procedure was approved by the Virginia Tech Institutional Animal Care and Use Committee.

### scRNA-Seq Library Construction

Cells cultured above were detached from the culture plate with trypsin-EDTA (0.25%) and washed with resuspension buffer (phosphate-buffered saline, 0.04% bovine serum albumin). Cells were filtered through a 40 µm strainer to remove cell clumps. Cell viability was determined to be 90% by trypan blue staining and hemocytometer counting. Two scRNA-seq libraries were constructed from satellite cells to increase the number of cells sequenced. The scRNA-seq libraries were constructed using the Chromium Single Cell 3ʹ Gel Bead-in-Emulsion (GEM), Library & Gel Bead Kit v3 (10x Genomics, Pleasanton, CA, United States). We adjusted cell concentration to 1,000 cells/μl with resuspension buffer, and loaded 12.8 µl of diluted cell suspension with a master mix of reverse transcription (RT) reagent, template switch oligo, and RT enzyme into a Chromium Chip B (10x Genomics). Single-cell GEM generation, barcoding, and reverse transcription were achieved by running the Chromium Chip B on the Chromium Controller (10x Genomics). Specifically, single cells, RT reagents, gel beads containing barcoded oligonucleotides, and oil were combined on a microfluidic chip to form reaction nanovesicles. Within each reaction nanovesicle, a single cell was lysed, the gel bead was dissolved to free the identically barcoded RT oligonucleotides, and polyadenylated mRNA was then reverse transcribed into cDNA. Thus, all cDNAs from the same cell would have the same barcode, which would allow the sequencing reads to be traced back to their single cells of origin. cDNA was amplified for 11 cycles. Based on an Agilent High Sensitivity TapeStation analysis, both scRNA-seq libraries contained a single peak of DNA between 300 and 700 bp, with an average fragment size of 440 bp.

### scRNA-Seq Sequencing and Data Analysis

Each scRNA-seq library was pair-end sequenced in a single cell flow lane on an Illumina HiSeq system at the Novogene-UC Davis Sequencing Center (Novogene, Sacramento, CA, United States). Sequencing reads were processed and analyzed using the 10x Genomics Cell Ranger 3.0.2 software, which was composed of different analysis pipelines (Zheng et al., 2017). Specifically, sequencing reads were de-multiplexed using the cellranger mkfastq pipeline and aligned to the bovine genome and transcriptome (Bos_taurus.ARS-UCD1.2) at default parameters using the cellranger count pipeline. Uniquely mapped sequences from each library were used for unique molecular identifiers (UMI) counting using the cellranger count pipeline. The output files from the cellranger counting of reads from two scRNA-seq libraries were combined using the cellranger aggr pipeline.

Cells were initially clustered by expression similarity using the graph-based clustering algorithm, which consisted of building a sparse nearest-neighbor graph followed by Louvain Modularity Optimization, an algorithm that sought to find highly-connected modules in the graph (Blondel et al., 2008). Cells were further clustered by an additional cluster-merging step, which included hierarchical clustering on the cluster-medoids in principal components analysis (PCA) space and merging pairs of sibling clusters if there were no genes differentially expressed between them (with B-H adjusted *p*-value below 0.05). The hierarchical clustering and merging was repeated until there were no more cluster-pairs to merge. Differential gene expression between cell clusters was identified using the quick and simple method sSeq and edgeR (Robinson and Smyth, 2007; Yu et al., 2013). All these analysis pipelines and algorithms were part of the Cell Ranger software and run using parameters recommended by 10X Genomics.

Cell clusters were annotated based on significantly enriched expression (with B-H adjusted *p*-value below 0.05) of marker genes of cell types. Cell clusters and gene expression data were visualized in the Loupe Cell Browser (10x Genomics). Gene ontology analysis was performed with the PANTHER classification system (Mi et al., 2013). Gene ontology terms with corrected *p*-values less than 0.05 were considered significantly enriched.

Trajectory inference was performed using the Monocle algorithm (version 2.20.0) (Trapnell et al., 2014; Qiu et al., 2017). Briefly, the output files of the cellranger count pipeline were read in to generate the count matrix using the Read10X function of Seurat 4 (Hao et al., 2021). A Seurat object was then built from the count matrix using the CreateSeuratObject function of Seurat. The Seurat object was converted to a CellDataSet modeled with the negative binomial distribution using the newimport function of Monocle. Genes with mean expression levels >0.1 were selected to define the state of cells. Following a dimensionality reduction using the reduceDimension function, cells were ordered along the trajectory using the orderCells function of Monocle.

Scripts and codes used to run the Cell Ranger, Seurat, and Monocle programs in this study were adopted from the manuals and websites for these programs and are listed in the Supplementary File S1.

## Results and Discussion

### Cultured Bovine Satellite Cells Are Heterogenous in Gene Expression

**FIGURE 1:**
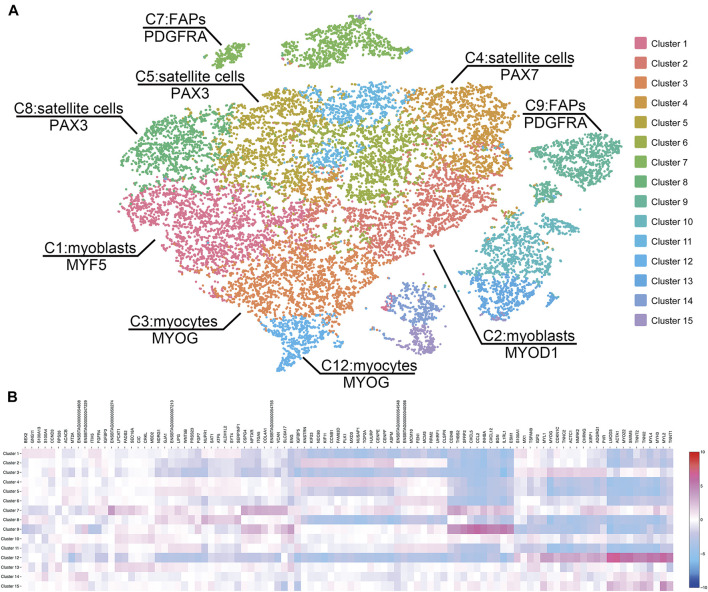
**(A)** Clustering of cells by the t-distributed stochastic neighbor embedding (t-SNE) algorithm. The 19,096 cultured bovine satellite cells are divided into 15 clusters. Cells close to each other have more similar gene expression patterns than those distant from each other. Cell types are inferred from significantly enriched expression of known cell markers. FAP, fibro-adipogenic progenitors. **(B)** Heatmap of transcriptome similarities between cell clusters. Rows represent cell clusters. Columns represent genes. Shown on top are representative genes. Numbers and colors on the right represent log2 fold changes relative to the median gene expression level across all clusters.

Sequencing of the two scRNA-seq libraries of bovine satellite cells in culture generated approximately 430 million reads per library. More than 85 and 50% of these reads were uniquely mapped to the bovine genome and transcriptome (bos_taurus.ars-ucd1.2), respectively (Table 1). Based on the Cell Ranger analyses, these reads represented the transcriptomes in more than 9,000 cells for each library (Table 1). The mean number of reads detected per cell was more than 43,000 for both libraries, and the median number of genes detected per cell was over 3,500 for both libraries (Table 1). These numbers met or exceeded the minimum requirements for a quality scRNA-seq analysis (Handley et al., 2015; Haque et al., 2017; Ziegenhain et al., 2017). Because the two scRNA-seq libraries were prepared from the same cells, sequencing data from the two libraries were combined to increase the total number of cells analyzed. Clustering the transcriptomes of 19,096 cells combined from the two scRNA-libraries revealed 15 cell clusters that differed in gene expression pattern (Figure 1, Supplementary File S2). These 15 clusters contained 300–2,500 cells, or 2–13% total cells analyzed, with cluster 1 being the largest cluster and cluster 15 being the smallest cluster (Table 2).

**TABLE 1:** Summary of scRNA-seq sequencing, mapping, and analysis.

Item	Library 1	Library 2	Aggregation
Number of reads	431,539,783	429,256,266	860,796,049
Valid barcodes	97.6%	97.4%	97.5%
Reads mapped uniquely to genome (bos_taurus.ars-ucd1.2)	85.5%	85.2%	85.4%
Reads mapped uniquely to transcriptome (bos_taurus.ars-ucd1.2)	50.8%	51.5%	51.1%
Fraction reads in cells	90.8%	89.1%	90.0%
Estimated number of cells	9,939	9,157	19,096
Mean reads per cell	43,418	46,877	43,528
Median genes per cell	3,559	3,764	3,609
Total genes detected	16,227	16,356	16,816

**TABLE 2:** Numbers and percentages of clustered cells.

	Number of cells	% Total cells
Cluster 1	2,493	13.1
Cluster 2	2,119	11.1
Cluster 3	2,034	10.7
Cluster 4	1,768	9.3
Cluster 5	1,641	8.6
Cluster 6	1,575	8.2
Cluster 7	1,298	6.8
Cluster 8	1,241	6.5
Cluster 9	1,080	5.7
Cluster 10	1,010	5.3
Cluster 11	845	4.4
Cluster 12	627	3.3
Cluster 13	602	3.2
Cluster 14	446	2.3
Cluster 15	317	1.7

### Cultured Bovine Satellite Cells Contain Subpopulations That Differ in Myogenic Stage and Proliferation Rate

**FIGURE 2:**
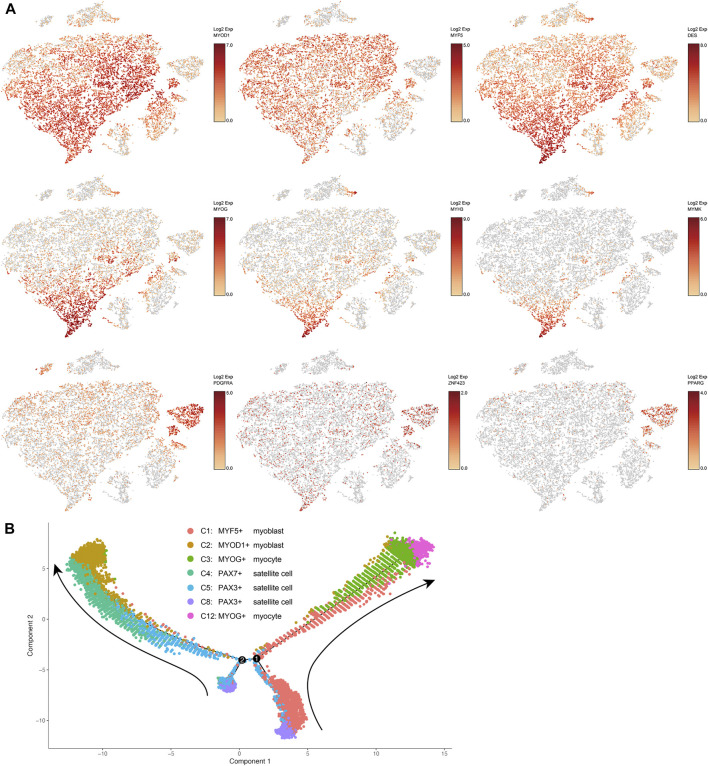
**(A)** t-SNE maps showing the expression levels of selected markers in different clusters of bovine satellite cells. MYOD1, MYF5, and DES are markers of myoblasts; MYOG, MYH3, and MYMK are markers of differentiated myoblasts or myocytes; PDGFRA, PPARG, and ZNF423 are markers of fibro-adipogenic progenitors and preadipocytes. **(B)** Trajectory inference analysis of myogenic cell clusters. The trajectory analysis was performed using Monocle. Cells in different clusters are represented by different colors. Cluster numbers and cell types correspond to those in Figure 1A. Arrows indicate the direction of trajectory. Black lines and numbers represent trajectory branches and branching points, respectively.

Based on the expression levels of marker genes (MYOD1, MYF5, and DES) of myoblasts (Pownall et al., 2002), clusters 1, 2, 3, and 12 were characterized as subsets of myoblasts, which are activated and proliferating satellite cells (Figures 1A, 2A). Expression of MYF5 mRNA in cluster 1, expression of MYOD1 mRNA in cluster 2, and expression of both MYF5 and MYOD1 mRNAs in cluster 3 were significantly enriched (Supplementary File S2, Figure 2A). PAX7 and PAX3 are markers of satellite cells (Maroto et al., 1997; Seale et al., 2000). Expression of PAX7 mRNA was significantly enriched in cluster 4, and PAX3 mRNA expression was significantly enriched in clusters 5 and 8 (Supplementary File S2). Because they were enriched with PAX7 or PAX3, clusters 4, 5, and 8 were determined to be subsets of satellite cells (Figure 1A).

MYOG is a master transcriptional regulator of myoblast differentiation (Pownall et al., 2002). MYOG mRNA expression was significantly enriched in clusters 3 and 12 (Supplementary File S2, Figure 2A). Besides MYOG, many muscle-specific genes such as MB, MYH3, MYL1, NEB, and STAC3 and several myoblast differentiation and fusion regulatory genes such as MEF2A, MEF2D and MYMK (Millay et al., 2013; Estrella et al., 2015) were upregulated in clusters 3 and 12 (Supplementary File S2, Figure 2A). These two clusters clearly contained differentiating or differentiated myoblasts, i.e., myocytes. Between clusters 3 and 12, more muscle-specific genes were upregulated in cluster 12 than in cluster 3, and the same muscle-specific genes were expressed at greater levels in cluster 12 than in cluster 3 (Supplementary File S2, Figure 2A). These differences suggest that myoblasts in cluster 12 were more terminally differentiated than those in cluster 3.

Gene ontology analyses of genes upregulated in each cluster indicated that cells in different clusters differed in function. For example, gene ontology analyses of genes upregulated in clusters 2 and 4 indicated that many of these genes were involved in the biological processes, cellular components, and molecular function related to DNA synthesis and cell cycle (Supplementary File S3), suggesting that cells in these clusters were undergoing active proliferation. Gene ontology analyses of genes upregulated in clusters 3 and 12 indicated that many of these genes were involved in the biological processes, cellular components, and molecular function related to mature skeletal muscle structure and contraction (Supplementary File S3), suggesting that cells in these two clusters were differentiating into functional muscle cells.

A trajectory inference analysis using the Monocle program (Trapnell et al., 2014) revealed the potential lineage relationships between the seven myogenic cell clusters, i.e., clusters 1–5, 8, and 12 (Figure 2B). This analysis suggested two trajectories along which the myogenic progenitors PAX3+ satellite cells transitioned toward myogenic differentiation. On one trajectory, PAX3+ satellite cells in cluster 8 committed to MYF5+ myoblasts in cluster 1, and these MYF5+ myoblasts then differentiated into MYOG+ myocytes in cluster 3, which then differentiated further into MYOG+ myocytes in cluster 12 (Figure 2B). On the other trajectory, PAX3+ satellite cells in cluster 8 first transitioned to a population of satellite cells in cluster 5 that had a different gene expression pattern from cells in cluster 8 but were still PAX3 positive; PAX3+ satellite cells in cluster 5 then became PAX7+ satellite cells in cluster 4, which were subsequently activated to become MYOD1+ myoblasts in cluster 2 (Figure 2B). Overall, this trajectory analysis further supports the conclusion earlier that cultured bovine satellite cells are composed of subsets of myogenic cells that differ in transcriptional and myogenic state.

### Cultured Bovine Satellite Cells Contain Subpopulations That May Be Intramuscular Preadipocytes

Platelet derived growth factor receptor alpha (PDGFRA) is established as a marker of the progenitor cells for intramuscular adipocytes, i.e., intramuscular fibro-adipogenic progenitor (FAP) cells, in mice and humans (Uezumi et al., 2010; Uezumi et al., 2014; Joe et al., 2010). The expression of PDGFRA mRNA was significantly higher in clusters 7 and 9 than in other clusters (Supplementary File S2 and Figure 2A). It is interesting to note that cells in cluster 9 were also enriched with peroxisome proliferator activated receptor gamma (PPARG) and zinc finger protein 423 (ZNF423) mRNAs, two transcriptional regulators of early adipogenesis (Tontonoz and Spiegelman, 2008; Gupta et al., 2010; Lefterova et al., 2014). Gene ontology analyses of genes upregulated in clusters 7 and 9 indicated active lipogenesis in cluster 9 (Supplementary File S3). Cells in neither cluster 7 nor cluster 9 expressed markers of mature adipocytes such as leptin (LEP) and adiponectin (ADIPOQ) (Supplementary File S2). These data together indicated that clusters 7 and 9 were FAPs, or intramuscular preadipocytes, with cluster 9 appearing to be more developed preadipocytes than cluster 7. Because cells in clusters 7 and 9 were not enriched with MYOD1, MYF5, DES, or MYOG, markers of myogenic cells, it remains to be determined if these FAP cells were derived from satellite cells during culture or accidently co-isolated with satellite cells from skeletal muscle.

### Cultured Bovine Satellite Cells Contain Subpopulations Whose Identities Remain to Be Determined

Compared to other clusters (Supplementary File S2), clusters 6, 10, 11, 13, 14, and 15 did not express significantly higher levels of markers of myogenic cells such as PAX3, PAX7, MYOD1, MYF5, and MYOG (Pownall et al., 2002); thus, these clusters were not myogenic cells. Skeletal muscle contains not only myogenic cells but also nonmyogenic cells such as endothelial cells, pericytes, smooth muscle cells, fibroblasts, and glial cells. However, none of clusters 6, 10, 11, 13, 14, and 15 appeared to be these nonmyogenic cells based on the expression levels of marker genes such as CDH5 and PECAM1 for endothelial cells (Elmentaite et al., 2021), NOTCH3 and MCAM for pericytes (Elmentaite et al., 2021), ACTA2 and MYH11 for smooth muscle cells (Kumar et al., 2017), COL1A and S100A4 for fibroblasts (Kumar et al., 2017), and FOXD3 and SOX10 for glial cells (Elmentaite et al., 2021) in these clusters (Supplementary File S2). It is possible that clusters 6, 10, 11, 13, 14, and 15 represented novel cell types in bovine skeletal muscle co-isolated with satellite cells or that they were inaccurately clustered by the computational program used.

## Conclusions

Results of this scRNA-seq study suggest that bovine satellite cells are possibly composed of subpopulations that differ in transcriptional status, proliferation rate, and myogenic potential. This notion is consistent with the conclusion from scRNA-seq studies of mouse satellite cells (Cho and Doles, 2017; van den Brink et al., 2017). Results of this study also suggest the presence of FAP cells in bovine skeletal muscle, although their origin remains to be determined. Because skeletal muscle growth and intramuscular fat are economically important traits in cattle, further characterization of the different subpopulations of satellite cells as well as FAPs in bovine skeletal muscle could lead to the development of new strategies to improve these traits or to identify DNA sequences and variants associated with these traits in cattle.

## Data Availability

The original contributions presented in the study are publicly available. This data can be found here: https://www.ncbi.nlm.nih.gov/geo/query/acc.cgi?acc=GSE184128.

## Glossary

ACTA2: actin alpha 2, smooth muscle
ADIPOQ: adiponectin
BP: biological process
CC: cellular component
CDH5: cadherin 5
COL1A: collagen type I alpha 1 chain
DES: desmin
FAPs: fibro-adipogenic progenitors
FDR: false discovery rate
FE: fold enrichment
FOXD3: forkhead box D3
GEM: gel bead-in-emulsion
GO: gene ontology
LEP: leptin
MB: myoglobin
MCAM: melanoma cell adhesion molecule
MEF: myocyte enhancer factor
MF: molecular function
MYF5: myogenic factor 5
MYH3: myosin heavy chain 3
MYH11: myosin heavy chain 11
MYL1: myosin light chain 1
MYMK: myomaker or myoblast fusion factor
MYOD1: myogenic differentiation 1
MYOG: myogenin
NEB: nebulin
NOTCH3: notch receptor 3
PAX3: paired box 3
PAX7: paired box 7
PDGFRA: platelet derived growth factor receptor alpha
PECAM1: platelet and endothelial cell adhesion molecule 1
PPARG: peroxisome proliferator activated receptor gamma
S100A4: S100 calcium binding protein A4
scRNA-seq: single-cell RNA sequencing
SOX10: SRY-box transcription factor 10
STAC3: SH3 and cysteine rich domain 3
UMI: unique molecular identifier
ZNF423: zinc finger protein 423
