# Supplementary material for: Single-cell RNA Sequencing Reveals Heterogeneity of Cultured Bovine Satellite Cells
Source: Front Genet. 2021 Oct 28;12:742077. doi: 10.3389/fgene.2021.742077 (PMC8580861; doi:10.3389/fgene.2021.742077)
Supplement: Supplementary file 2 [file DataSheet1.pdf]

```
#=============cellranger step============
#==========S1_demultiplexed Fastq file===

# fastq files were demultiplexed by Novogene
# index J11 SI-GA-A8    GCATCTCC,TGTAAGGT,CTGCGATG,AACGTCAA
            J22 SI-GA-A9        TCTTAAAG,CGAGGCTC,GTCCTTCT,AAGACGGA

#==============S1.1_build reference======

cellranger mkref --genome=Bos_taurus.ARS-UCD1.2
--fasta=Bos_taurus.ARS-UCD1.2.dna.toplevel.fa
--genes=Bos_taurus.ARS-UCD1.2.95.filtered.gtf

#==============S2_count=================

cellranger count  --id=J11 --fastqs=/home/pengcheng/fastq/J11/
--transcriptome=/home/pengcheng/ref/Bos_taurus.ARS-UCD1.2/
cellranger count  --id=J22 --fastqs=/home/pengcheng/fastq/J22/
--transcriptome=/home/pengcheng/ref/Bos_taurus.ARS-UCD1.2/

#==============S3_aggreate===================

cellranger aggr --id=J11J22 --csv=J11J22_libraries.csv --normalize=mapped

#J11J22_libraries.csv
#library_id     molecule_h5
#J11    /home/pengcheng/J11/outs/molecule_info.h5
#J22    /home/pengcheng/J22/outs/molecule_info.h5

#================R_step(Seurat&Monocle)============================
library(Seurat)
library(monocle)
library(dplyr)
#================Create Seurat object==============================

bsc<- Read10X(data.dir = "/Users/pengchenglyu/scRNA/J1122/")
bsc <- CreateSeuratObject(counts = bsc, project = "bsc_10X", min.cells = 3,
min.features = 200)

cluster <- read.csv2(file =
"/Users/pengchenglyu/scRNA/J1122/analysis/clustering/graphclust/clusters.csv" , sep
= ",")
cluster_myogenic <- subset(x =cluster , Cluster == "12" | Cluster == "3" |
                             Cluster == "1" | Cluster == "2"  |
                             Cluster == "4" |Cluster == "5" | Cluster == "8" )
myogenic_barcode <- c(cluster_myogenic$Barcode)
pbmc <- subset(x = bsc , cells = myogenic_barcode)
pbmc@meta.data$cluster <- as.factor( cluster_myogenic$Cluster)

pbmc[["percent.mt"]] <- PercentageFeatureSet(pbmc, pattern =
```

```
"(ND[1-6]|COX[1-3]|ATP6|ATP8|CYTB|ND4L)")

#================ Monocle ==========================================
newimport <- function(otherCDS, import_all = FALSE) {
  if(class(otherCDS)[1] == 'Seurat') {
    requireNamespace("Seurat")
    data <- otherCDS@assays$RNA@counts

    if(class(data) == "data.frame") {
      data <- as(as.matrix(data), "sparseMatrix")
    }

    pd <- tryCatch( {
      pd <- new("AnnotatedDataFrame", data = otherCDS@meta.data)
      pd
    },
    #warning = function(w) { },
    error = function(e) {
      pData <- data.frame(cell_id = colnames(data), row.names = colnames(data))
      pd <- new("AnnotatedDataFrame", data = pData)

      message("This Seurat object doesn't provide any meta data");
      pd
    })

    # remove filtered cells from Seurat
    if(length(setdiff(colnames(data), rownames(pd))) > 0) {
      data <- data[, rownames(pd)]
    }

    fData <- data.frame(gene_short_name = row.names(data), row.names =
row.names(data))
    fd <- new("AnnotatedDataFrame", data = fData)
    lowerDetectionLimit <- 0

    if(all(data == floor(data))) {
      expressionFamily <- negbinomial.size()
    } else if(any(data < 0)){
      expressionFamily <- uninormal()
    } else {
      expressionFamily <- tobit()
    }

    valid_data <- data[, row.names(pd)]

    monocle_cds <- newCellDataSet(data,
                                  phenoData = pd,
                                  featureData = fd,
                                  lowerDetectionLimit=lowerDetectionLimit,
                                  expressionFamily=expressionFamily)
```

```
    if(import_all) {
      if("Monocle" %in% names(otherCDS@misc)) {
        otherCDS@misc$Monocle@auxClusteringData$seurat <- NULL
        otherCDS@misc$Monocle@auxClusteringData$scran <- NULL

        monocle_cds <- otherCDS@misc$Monocle
        mist_list <- otherCDS

      } else {
        # mist_list <- list(ident = ident)
        mist_list <- otherCDS
      }
    } else {
      mist_list <- list()
    }

    if(1==1) {
      var.genes <- setOrderingFilter(monocle_cds, otherCDS@assays$RNA@var.features)

    }
    monocle_cds@auxClusteringData$seurat <- mist_list

  } else if (class(otherCDS)[1] == 'SCESet') {
    requireNamespace("scater")

    message('Converting the exprs data in log scale back to original scale ...')
    data <- 2^otherCDS@assayData$exprs - otherCDS@logExprsOffset

    fd <- otherCDS@featureData
    pd <- otherCDS@phenoData
    experimentData = otherCDS@experimentData
    if("is.expr" %in% slotNames(otherCDS))
      lowerDetectionLimit <- otherCDS@is.expr
    else
      lowerDetectionLimit <- 1

    if(all(data == floor(data))) {
      expressionFamily <- negbinomial.size()
    } else if(any(data < 0)){
      expressionFamily <- uninormal()
    } else {
      expressionFamily <- tobit()
    }

    if(import_all) {
      # mist_list <- list(iotherCDS@sc3,
      #                   otherCDS@reducedDimension)
      mist_list <- otherCDS
```

```
    } else {
      mist_list <- list()
    }

    monocle_cds <- newCellDataSet(data,
                                  phenoData = pd,
                                  featureData = fd,
                                  lowerDetectionLimit=lowerDetectionLimit,
                                  expressionFamily=expressionFamily)
    # monocle_cds@auxClusteringData$sc3 <- otherCDS@sc3
    # monocle_cds@auxOrderingData$scran <- mist_list

    monocle_cds@auxOrderingData$scran <- mist_list

  } else {
    stop('the object type you want to export to is not supported yet')
  }

  return(monocle_cds)
}  # the function used to read Seurat4 object

cds <- newimport(pbmc) # import S4 object

#===================prepare data for dimension reduction=====

cds <- estimateSizeFactors(cds)
cds <- estimateDispersions(cds)

#===================select high expression gene==============

disp_table <- dispersionTable(cds)
unsup_clustering_genes <- subset(disp_table,mean_expression >= 0.1)  #pick high
expression genes
cds <- setOrderingFilter(cds , unsup_clustering_genes$gene_id)

#===================dimension reduction & order cells========
cds <- reduceDimension(cds, max_components = 2, method = 'DDRTree')
cds <- orderCells(cds)
p1 <- plot_cell_trajectory(cds , color_by = "cluster")
```
